# 8-(2-Hy­droxy­phen­yl)-1,3-dimethyl-1*H*-purine-2,6(3*H*,7*H*)-dione

**DOI:** 10.1107/S1600536811032417

**Published:** 2011-08-17

**Authors:** Irvin Booysen, Thulani Hlela, Muhammed Ismail, Thomas Gerber, Eric Hosten, Richard Betz

**Affiliations:** a University of Kwazulu-Natal, School of Chemistry, Private Bag X01, Scottsville 3209, Pietermaritzburg, South Africa; b Nelson Mandela Metropolitan University, Summerstrand Campus, Department of Chemistry, University Way, Summerstrand, PO Box 77000, Port Elizabeth 6031, South Africa

## Abstract

The title compound, C_13_H_12_N_4_O_3_, is an imidazole derivative featuring an annealed purine ring system. The benzimidazole-inspired moiety is essentially planar (r.m.s. of all fitted non-H atoms = 0.0205 Å). An intra­molecular O—H⋯N hydrogen bond occurs. In the crystal, inter­molecular N—H⋯O and C—H⋯O hydrogen bonds are observed, which connect the mol­ecules into chains along [110]. The shortest centroid–centroid distance between two aromatic systems is 3.7771 (11) Å.

## Related literature

For the crystal structure of benzimidazole, see: Krawczyk & Gdaniec (2005 ▶). For the crystal structure of hypoxanthinium nitrate monohydrate as an example of an oxopurine compound, see: Schmalle *et al.* (1990 ▶). For graph-set analysis of hydrogen bonds, see: Etter *et al.* (1990 ▶); Bernstein *et al.* (1995 ▶). For puckering analysis, see: Cremer & Pople (1975 ▶). For general information about the chelate effect in coordination chemistry, see: Gade (1998 ▶).
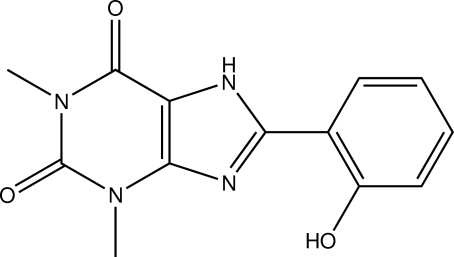


## Experimental

### 

#### Crystal data

C_13_H_12_N_4_O_3_
*M* *_r_* = 272.27
Monoclinic, 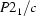

*a* = 8.6418 (5) Å
*b* = 5.9415 (3) Å
*c* = 23.4475 (10) Å
β = 91.275 (2)°
*V* = 1203.62 (11) Å^3^
*Z* = 4
Mo *K*α radiation
μ = 0.11 mm^−1^
*T* = 200 K
0.41 × 0.10 × 0.05 mm

#### Data collection

Bruker APEXII CCD diffractometer
Absorption correction: multi-scan (*SADABS*; Bruker, 2008 ▶) *T*_min_ = 0.860, *T*_max_ = 1.000
10068 measured reflections
2975 independent reflections
1752 reflections with *I* > 2σ(*I*)
*R*_int_ = 0.043

#### Refinement

*R*[*F* ^2^ > 2σ(*F* ^2^)] = 0.049
*wR*(*F* ^2^) = 0.124
*S* = 1.01
2975 reflections
188 parameters
H atoms treated by a mixture of independent and constrained refinement
Δρ_max_ = 0.25 e Å^−3^
Δρ_min_ = −0.21 e Å^−3^

### 

Data collection: *APEX2* (Bruker, 2010 ▶); cell refinement: *SAINT* (Bruker, 2010 ▶); data reduction: *SAINT*; program(s) used to solve structure: *SHELXS97* (Sheldrick, 2008 ▶); program(s) used to refine structure: *SHELXL97* (Sheldrick, 2008 ▶); molecular graphics: *ORTEP-3* (Farrugia, 1997 ▶) and *Mercury* (Macrae *et al.*, 2008 ▶); software used to prepare material for publication: *SHELXL97* and *PLATON* (Spek, 2009 ▶).

## Supplementary Material

Crystal structure: contains datablock(s) I, global. DOI: 10.1107/S1600536811032417/si2369sup1.cif

Supplementary material file. DOI: 10.1107/S1600536811032417/si2369Isup2.cdx

Structure factors: contains datablock(s) I. DOI: 10.1107/S1600536811032417/si2369Isup3.hkl

Supplementary material file. DOI: 10.1107/S1600536811032417/si2369Isup4.cml

Additional supplementary materials:  crystallographic information; 3D view; checkCIF report

## Figures and Tables

**Table 1:** Hydrogen-bond geometry (Å, °)

*D*—H⋯*A*	*D*—H	H⋯*A*	*D*⋯*A*	*D*—H⋯*A*
O3—H3⋯N2	0.84	1.86	2.611 (2)	148
N1—H71⋯O1^i^	0.97 (2)	1.78 (2)	2.746 (2)	175.4 (19)
C9—H9⋯O1^i^	0.95	2.37	3.294 (2)	164
C5—H5*A*⋯O3^ii^	0.98	2.58	3.234 (2)	124

Symmetry codes: (i) 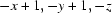; (ii) 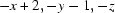.

**Fig. 1.**
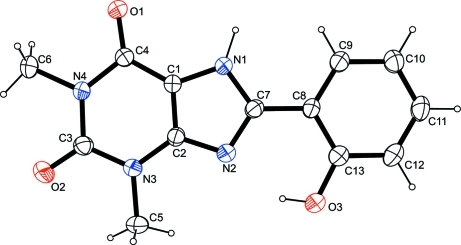
The molecular structure of the title compound, with atom labels and anisotropic displacement ellipsoids (drawn at 50% probability level).

**Fig. 2.**
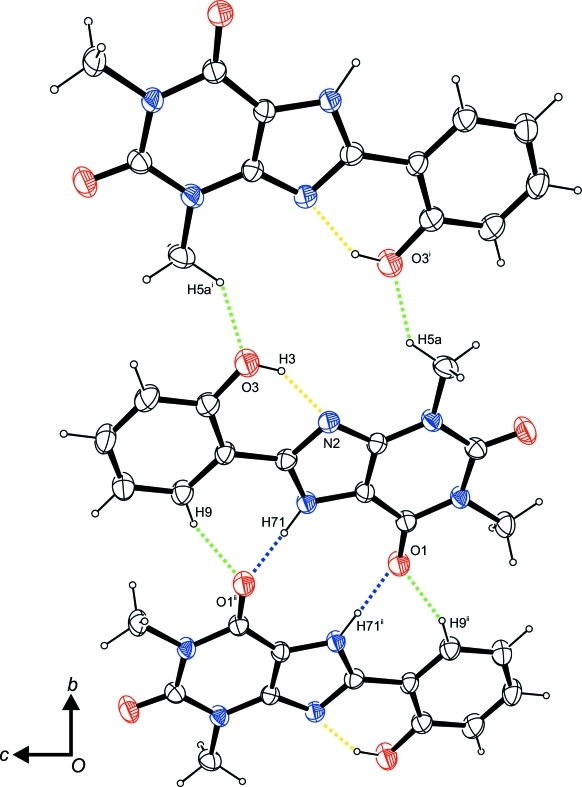
Intermolecular contacts, viewed along [-1 0 0]. Symmetry operators: i -x + 2, -y - 1, -z; ii -x + 1, -y + 1, -z.

**Fig. 3.**
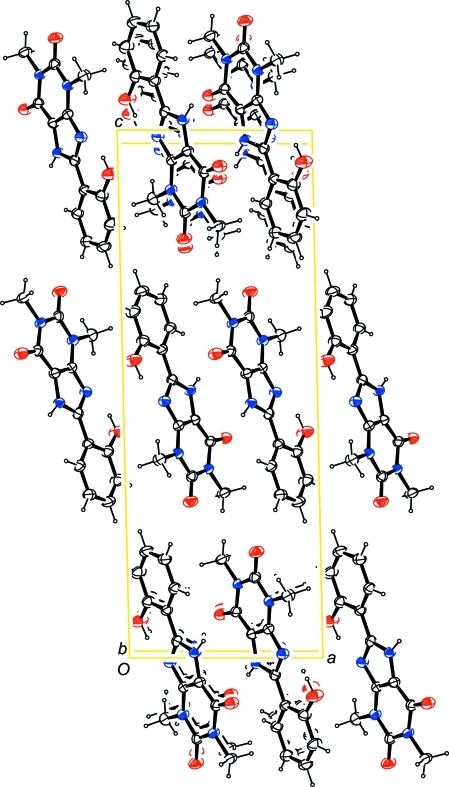
Molecular packing of the title compound, viewed along [0 1 0] (anisotropic displacement ellipsoids drawn at 50% probability level).

## supplementary crystallographic information

### Comment

Chelate ligands have found widespread use in coordination chemistry due to the enhanced thermodynamic stability of resultant coordination compounds in relation to metal complexes exclusively applying comparable monodentate ligands (Gade 1998). Combining different sets of donor atoms in one chelate ligand molecule, a probe for testing and accomodating metal centers of different Lewis acidities is at hand. To enable comparative studies with envisioned coordination compounds, we determined the crystal structure of the title compound. The crystal structure of benzimidazole has been reported various times in the literature (*e.g.* Krawczyk & Gdaniec, 2005). In addition, several oxopurine derivatives have been the topic of crystal structure determinations (*e.g.* Schmalle *et al.*, 1990).

The molecule features a benzimidazole-inspired backbone comprised of a 6-amino-1,3-dimethylpyrimidine-2,4(1*H*,3*H*)-dione moiety which is annealed to a five-membered aromatic ring. This part of the molecule is essentially planar (r.m.s. of its fitted non-hydrogen atoms = 0.0205 Å). The small puckering amplitude (τ = 1.6 °) of the six-membered heterocycle precludes a conformation analysis (Cremer & Pople, 1975). The least-squares planes defined by the atoms of the phenyl ring on the one hand and the benzimidazole-type ring system on the other hand enclose an angle of 0.45 (10) ° (Fig. 1). Both C–N–C angles in the five-membered heterocycle are similar in value with 106.25 (15)° and 104.41 (15)°, with the smaller value found on the non-protonated nitrogen atom. However, these angles are smaller in value than the corresponding ones in hypoxanthinium nitrate monohydrate – invariably above 108 ° – where both nitrogen atoms bear a hydrogen atom (Schmalle *et al.*, 1990).

In the crystal structure, intra- as well as intermolecular hydrogen bonds and C–H···O contacts whose range falls by more than 0.1 Å below the sum of van-der-Waals radii of atoms participating are observed. While the intramolecular hydrogen bonds are exclusively made up by the proton of the hydroxyl group as donor and the non-protonated nitrogen atom of the five-membered heterocycle, intermolecular hydrogen bonds are solely apparent between the amino group and one of the double-bonded oxygen atoms (Table 1). The C–H···O contacts can be separated in two groups: while one of the nitrogen-bound methyl groups forms a C–H···O contact involving the oxygen atom of the hydroxyl group, one of the aromatic C–H groups acts as donor for the double-bonded oxygen atom that is already part of the N–H···O type hydrogen bonds. In terms of graph-set analysis (Etter *et al.*, 1990; Bernstein *et al.*, 1995), the descriptor for the classical hydrogen bonds is *S*(6)*R*^2^_2_(10) on the unitary level. For the C–H···O contacts, a *R*^2^_2_(16)*R*^2^_2_(18) descriptor on the same level is needed for description. In total, the molecules are connected to chains along [1 1 0]. The shortest intercentroid distance between two aromatic systems was measured at 3.7771 (11) Å (Fig. 2).

The packing of the title compound in the crystal is shown in Figure 3.

### Experimental

The title compound was prepared by reacting 6-amino-1,3-dimethyl-5-[(*E*)- 2-(hydroxy)benzylideneamino]pyrimidine-2,4-(1*H*,3*H*)-dione and NH_4_VO_3_ in methanol. A bright yellow precipitate was filtered, washed with methanol and dried under reduced pressure. Single-crystals suitable for the X-ray diffraction study were obtained by recrystallization from ethanol-dichlormethane (*v*:*v* = 1:1) mixture which was left in a fridge for several days.

### Refinement

Carbon-bound H atoms were placed in calculated positions (C—H 0.95 Å) and were included in the refinement in the riding model approximation, with *U*(H) set to 1.2*U*_eq_(C). The H atoms of the methyl groups were allowed to rotate with a fixed angle around the C—C bond to best fit the experimental electron density (HFIX 137 in the *SHELX* program suite (Sheldrick, 2008)), with *U*(H) set to 1.5*U*_eq_(C). The nitrogen-bound H atom was located on a difference Fourier map and refined freely.

### Crystal data

**Table:**

C_13_H_12_N_4_O_3_	*F*(000) = 568
*M**_r_* = 272.27	*D*_x_ = 1.502 Mg m^−^^3^
Monoclinic, *P*2_1_/*c*	Mo *K*α radiation, λ = 0.71073 Å
Hall symbol: -P 2ybc	Cell parameters from 1955 reflections
*a* = 8.6418 (5) Å	θ = 2.4–27.2°
*b* = 5.9415 (3) Å	µ = 0.11 mm^−^^1^
*c* = 23.4475 (10) Å	*T* = 200 K
β = 91.275 (2)°	Platelet, yellow
*V* = 1203.62 (11) Å^3^	0.41 × 0.10 × 0.05 mm
*Z* = 4	

### Data collection

**Table:**

Bruker APEXII CCD diffractometer	2975 independent reflections
Radiation source: fine-focus sealed tube	1752 reflections with *I* > 2σ(*I*)
graphite	*R*_int_ = 0.043
φ and ω scans	θ_max_ = 28.3°, θ_min_ = 2.4°
Absorption correction: multi-scan (*SADABS*; Bruker, 2008)	*h* = −11→11
*T*_min_ = 0.860, *T*_max_ = 1.000	*k* = −7→6
10068 measured reflections	*l* = −31→27

### Refinement

**Table:**

Refinement on *F*^2^	Primary atom site location: structure-invariant direct methods
Least-squares matrix: full	Secondary atom site location: difference Fourier map
*R*[*F*^2^ > 2σ(*F*^2^)] = 0.049	Hydrogen site location: inferred from neighbouring sites
*wR*(*F*^2^) = 0.124	H atoms treated by a mixture of independent and constrained refinement
*S* = 1.01	*w* = 1/[σ^2^(*F*_o_^2^) + (0.0584*P*)^2^ + 0.0815*P*] where *P* = (*F*_o_^2^ + 2*F*_c_^2^)/3
2975 reflections	(Δ/σ)_max_ < 0.001
188 parameters	Δρ_max_ = 0.25 e Å^−^^3^
0 restraints	Δρ_min_ = −0.21 e Å^−^^3^

### Fractional atomic coordinates and isotropic or equivalent isotropic displacement parameters (Å^2^)

**Table:**

	*x*	*y*	*z*	*U*_iso_*/*U*_eq_	
O1	0.47991 (16)	0.4606 (2)	0.07866 (5)	0.0356 (4)	
O2	0.66823 (16)	−0.0720 (2)	0.20267 (6)	0.0390 (4)	
O3	0.94790 (18)	−0.3204 (3)	−0.06725 (6)	0.0454 (4)	
H3	0.9105	−0.2923	−0.0353	0.068*	
N1	0.65931 (17)	0.2091 (3)	−0.01367 (6)	0.0260 (4)	
H71	0.615 (2)	0.330 (4)	−0.0365 (9)	0.049 (7)*	
N2	0.79184 (18)	−0.1028 (3)	0.00928 (6)	0.0273 (4)	
N3	0.73063 (17)	−0.1106 (3)	0.10978 (6)	0.0269 (4)	
N4	0.57335 (17)	0.1913 (3)	0.13977 (6)	0.0259 (4)	
C1	0.6392 (2)	0.1685 (3)	0.04385 (7)	0.0247 (4)	
C2	0.7221 (2)	−0.0211 (3)	0.05591 (7)	0.0238 (4)	
C3	0.6584 (2)	−0.0024 (3)	0.15408 (8)	0.0267 (4)	
C4	0.5573 (2)	0.2871 (3)	0.08562 (7)	0.0254 (4)	
C5	0.8231 (2)	−0.3115 (3)	0.12198 (8)	0.0323 (5)	
H5A	0.8296	−0.4032	0.0874	0.048*	
H5B	0.7742	−0.3993	0.1520	0.048*	
H5C	0.9274	−0.2668	0.1347	0.048*	
C6	0.4923 (2)	0.3034 (4)	0.18658 (8)	0.0342 (5)	
H6A	0.5389	0.4513	0.1938	0.051*	
H6B	0.5012	0.2112	0.2212	0.051*	
H6C	0.3827	0.3221	0.1759	0.051*	
C7	0.7514 (2)	0.0418 (3)	−0.03268 (8)	0.0254 (4)	
C8	0.8012 (2)	0.0162 (3)	−0.09136 (8)	0.0258 (4)	
C9	0.7563 (2)	0.1677 (3)	−0.13407 (8)	0.0307 (5)	
H9	0.6913	0.2908	−0.1248	0.037*	
C10	0.8043 (2)	0.1426 (4)	−0.18967 (8)	0.0352 (5)	
H10	0.7732	0.2476	−0.2182	0.042*	
C11	0.8985 (2)	−0.0377 (4)	−0.20303 (8)	0.0383 (5)	
H11	0.9316	−0.0568	−0.2411	0.046*	
C12	0.9443 (2)	−0.1892 (4)	−0.16178 (8)	0.0391 (5)	
H12	1.0093	−0.3115	−0.1716	0.047*	
C13	0.8968 (2)	−0.1664 (3)	−0.10562 (8)	0.0316 (5)	

### Atomic displacement parameters (Å^2^)

**Table:**

	*U*^11^	*U*^22^	*U*^33^	*U*^12^	*U*^13^	*U*^23^
O1	0.0444 (8)	0.0353 (8)	0.0272 (8)	0.0189 (7)	0.0052 (6)	0.0019 (6)
O2	0.0474 (9)	0.0435 (9)	0.0265 (8)	0.0104 (7)	0.0069 (6)	0.0106 (7)
O3	0.0634 (10)	0.0420 (10)	0.0311 (8)	0.0278 (8)	0.0109 (7)	0.0036 (7)
N1	0.0290 (9)	0.0272 (9)	0.0219 (8)	0.0080 (7)	0.0029 (6)	0.0010 (7)
N2	0.0320 (9)	0.0256 (9)	0.0245 (8)	0.0059 (7)	0.0016 (7)	−0.0002 (7)
N3	0.0296 (9)	0.0270 (9)	0.0240 (8)	0.0059 (7)	0.0022 (6)	0.0037 (7)
N4	0.0284 (8)	0.0283 (9)	0.0212 (8)	0.0062 (7)	0.0047 (6)	0.0010 (7)
C1	0.0278 (10)	0.0251 (10)	0.0212 (9)	0.0041 (8)	0.0024 (7)	0.0008 (8)
C2	0.0248 (9)	0.0243 (10)	0.0224 (9)	0.0006 (8)	0.0029 (7)	−0.0011 (8)
C3	0.0261 (10)	0.0269 (11)	0.0270 (10)	0.0011 (8)	0.0028 (8)	0.0032 (8)
C4	0.0252 (10)	0.0270 (11)	0.0241 (10)	0.0034 (9)	0.0015 (8)	0.0006 (8)
C5	0.0356 (11)	0.0274 (11)	0.0339 (11)	0.0090 (9)	0.0015 (8)	0.0052 (9)
C6	0.0396 (11)	0.0399 (13)	0.0233 (10)	0.0084 (10)	0.0088 (8)	0.0003 (9)
C7	0.0246 (10)	0.0257 (10)	0.0257 (10)	0.0038 (8)	0.0012 (7)	−0.0019 (8)
C8	0.0274 (10)	0.0282 (10)	0.0219 (9)	0.0036 (8)	0.0031 (7)	−0.0025 (8)
C9	0.0309 (10)	0.0341 (12)	0.0271 (10)	0.0063 (9)	0.0028 (8)	−0.0014 (9)
C10	0.0370 (11)	0.0425 (13)	0.0261 (10)	0.0046 (10)	0.0019 (9)	0.0018 (10)
C11	0.0439 (12)	0.0475 (14)	0.0239 (11)	0.0060 (11)	0.0067 (9)	−0.0053 (10)
C12	0.0458 (13)	0.0388 (13)	0.0330 (12)	0.0124 (11)	0.0090 (9)	−0.0070 (10)
C13	0.0352 (11)	0.0308 (11)	0.0289 (11)	0.0058 (9)	0.0026 (8)	−0.0012 (9)

### Geometric parameters (Å, °)

**Table:**

O1—C4	1.237 (2)	C5—H5A	0.9800
O2—C3	1.213 (2)	C5—H5B	0.9800
O3—C13	1.351 (2)	C5—H5C	0.9800
O3—H3	0.8400	C6—H6A	0.9800
N1—C7	1.355 (2)	C6—H6B	0.9800
N1—C1	1.385 (2)	C6—H6C	0.9800
N1—H71	0.97 (2)	C7—C8	1.459 (2)
N2—C7	1.346 (2)	C8—C9	1.395 (3)
N2—C2	1.351 (2)	C8—C13	1.409 (3)
N3—C2	1.371 (2)	C9—C10	1.385 (3)
N3—C3	1.382 (2)	C9—H9	0.9500
N3—C5	1.461 (2)	C10—C11	1.385 (3)
N4—C4	1.396 (2)	C10—H10	0.9500
N4—C3	1.402 (2)	C11—C12	1.373 (3)
N4—C6	1.474 (2)	C11—H11	0.9500
C1—C2	1.361 (3)	C12—C13	1.394 (3)
C1—C4	1.410 (3)	C12—H12	0.9500
			
C13—O3—H3	109.5	H5B—C5—H5C	109.5
C7—N1—C1	106.25 (15)	N4—C6—H6A	109.5
C7—N1—H71	126.1 (13)	N4—C6—H6B	109.5
C1—N1—H71	127.6 (13)	H6A—C6—H6B	109.5
C7—N2—C2	104.41 (15)	N4—C6—H6C	109.5
C2—N3—C3	119.72 (16)	H6A—C6—H6C	109.5
C2—N3—C5	121.03 (15)	H6B—C6—H6C	109.5
C3—N3—C5	119.14 (15)	N2—C7—N1	111.87 (15)
C4—N4—C3	126.42 (15)	N2—C7—C8	123.05 (16)
C4—N4—C6	116.97 (15)	N1—C7—C8	125.08 (16)
C3—N4—C6	116.61 (15)	C9—C8—C13	118.81 (17)
C2—C1—N1	105.66 (16)	C9—C8—C7	121.77 (17)
C2—C1—C4	122.63 (17)	C13—C8—C7	119.42 (17)
N1—C1—C4	131.69 (17)	C10—C9—C8	121.44 (18)
N2—C2—C1	111.80 (16)	C10—C9—H9	119.3
N2—C2—N3	126.19 (17)	C8—C9—H9	119.3
C1—C2—N3	122.00 (16)	C9—C10—C11	119.09 (19)
O2—C3—N3	121.57 (17)	C9—C10—H10	120.5
O2—C3—N4	122.01 (17)	C11—C10—H10	120.5
N3—C3—N4	116.43 (16)	C12—C11—C10	120.61 (18)
O1—C4—N4	120.16 (16)	C12—C11—H11	119.7
O1—C4—C1	127.08 (17)	C10—C11—H11	119.7
N4—C4—C1	112.74 (16)	C11—C12—C13	121.01 (19)
N3—C5—H5A	109.5	C11—C12—H12	119.5
N3—C5—H5B	109.5	C13—C12—H12	119.5
H5A—C5—H5B	109.5	O3—C13—C12	117.67 (18)
N3—C5—H5C	109.5	O3—C13—C8	123.27 (17)
H5A—C5—H5C	109.5	C12—C13—C8	119.05 (18)
			
C7—N1—C1—C2	0.44 (19)	C2—C1—C4—O1	179.62 (19)
C7—N1—C1—C4	179.0 (2)	N1—C1—C4—O1	1.2 (3)
C7—N2—C2—C1	0.3 (2)	C2—C1—C4—N4	1.1 (3)
C7—N2—C2—N3	−179.25 (17)	N1—C1—C4—N4	−177.26 (18)
N1—C1—C2—N2	−0.5 (2)	C2—N2—C7—N1	0.0 (2)
C4—C1—C2—N2	−179.25 (17)	C2—N2—C7—C8	−179.54 (17)
N1—C1—C2—N3	179.11 (16)	C1—N1—C7—N2	−0.3 (2)
C4—C1—C2—N3	0.4 (3)	C1—N1—C7—C8	179.23 (17)
C3—N3—C2—N2	177.08 (17)	N2—C7—C8—C9	179.44 (17)
C5—N3—C2—N2	0.9 (3)	N1—C7—C8—C9	0.0 (3)
C3—N3—C2—C1	−2.5 (3)	N2—C7—C8—C13	−0.4 (3)
C5—N3—C2—C1	−178.64 (17)	N1—C7—C8—C13	−179.78 (18)
C2—N3—C3—O2	−177.22 (18)	C13—C8—C9—C10	−0.4 (3)
C5—N3—C3—O2	−1.0 (3)	C7—C8—C9—C10	179.85 (18)
C2—N3—C3—N4	2.8 (2)	C8—C9—C10—C11	0.3 (3)
C5—N3—C3—N4	179.07 (16)	C9—C10—C11—C12	−0.3 (3)
C4—N4—C3—O2	178.72 (17)	C10—C11—C12—C13	0.3 (3)
C6—N4—C3—O2	−1.7 (3)	C11—C12—C13—O3	−179.3 (2)
C4—N4—C3—N3	−1.3 (3)	C11—C12—C13—C8	−0.4 (3)
C6—N4—C3—N3	178.23 (16)	C9—C8—C13—O3	179.20 (18)
C3—N4—C4—O1	−179.24 (17)	C7—C8—C13—O3	−1.0 (3)
C6—N4—C4—O1	1.2 (3)	C9—C8—C13—C12	0.4 (3)
C3—N4—C4—C1	−0.6 (3)	C7—C8—C13—C12	−179.82 (18)
C6—N4—C4—C1	179.82 (16)		

### Hydrogen-bond geometry (Å, °)

**Table:**

*D*—H···*A*	*D*—H	H···*A*	*D*···*A*	*D*—H···*A*
O3—H3···N2	0.84	1.86	2.611 (2)	148.
N1—H71···O1^i^	0.97 (2)	1.78 (2)	2.746 (2)	175.4 (19)
C9—H9···O1^i^	0.95	2.37	3.294 (2)	164.
C5—H5A···O3^ii^	0.98	2.58	3.234 (2)	124.

Symmetry codes: (i) −*x*+1, −*y*+1, −*z*; (ii) −*x*+2, −*y*−1, −*z*.
